# Intravascular Administration of Mannitol for Acute Kidney Injury Prevention: A Systematic Review and Meta-Analysis

**DOI:** 10.1371/journal.pone.0085029

**Published:** 2014-01-14

**Authors:** Bo Yang, Jing Xu, Fengying Xu, Zui Zou, Chaoyang Ye, Changlin Mei, Zhiguo Mao

**Affiliations:** 1 Kidney Institute of Chinese People's Liberation Army, Division of Nephrology, Changzheng Hospital, Second Military Medical University, Shanghai, China; 2 Division of Anesthesiology, Changzheng Hospital, Second Military Medical University, Shanghai, China; Center for Molecular Biotechnology, Italy

## Abstract

**Background:**

The effects of mannitol administration on acute kidney injury (AKI) prevention remain uncertain, as the results from clinical studies were conflicting. Due to the lack of strong evidence, the KDIGO Guideline for AKI did not propose completely evidence-based recommendations on this issue.

**Methods:**

We searched PubMed, EMBASE, clinicaltrials.gov and Cochrane Controlled Trials Register. Randomized controlled trials on adult patients at increased risk of AKI were considered on the condition that they compared the effects of intravascular administration of mannitol plus expansion of intravascular volume with expansion of intravascular volume alone. We calculated pooled risk ratios, numbers needed to treat and mean differences with 95% confidence intervals for dichotomous data and continuous data, respectively.

**Results:**

Nine trials involving 626 patients were identified. Compared with expansion of intravascular volume alone, mannitol infusion for AKI prevention in high-risk patients can not reduce the serum creatinine level (MD 1.63, 95% CI −6.02 to 9.28). Subgroup analyses demonstrated that serum creatinine level is negatively affected by the use of mannitol in patients undergoing an injection of radiocontrast agents (MD 17.90, 95% CI 8.56 to 27.24). Mannitol administration may reduce the incidence of acute renal failure or the need of dialysis in recipients of renal transplantation (RR 0.34, 95% CI 0.21 to 0.57, NNT 3.03, 95% CI 2.17 to 5.00). But similar effects were not found in patients at high AKI risk, without receiving renal transplantation (RR 0.29, 95% CI 0.01 to 6.60).

**Conclusions:**

Intravascular administration of mannitol does not convey additional beneficial effects beyond adequate hydration in the patients at increased risk of AKI. For contrast-induced nephropathy, the use of mannitol is even detrimental. Further research evaluating the efficiency of mannitol infusions in the recipients of renal allograft should be undertaken.

## Introduction

Acute kidney injury (AKI) is defined as an abrupt decrease of renal function. It is a broad clinical syndrome encompassing various etiologies, including sepsis, dehydration, cardiac surgery (especially with cardiopulmonary bypass (CPB)), radiocontrast agents and so on.[1]–[3] AKI is associated with prolonged length of stay, increased mortality, and high health-care costs.[4]–[6] Early recognition and management of the patients at increased risk of AKI are paramount.

As a clinical strategy of AKI prevention, the use of diuretics has been well studied. Practice guideline[1] and comprehensive meta-analyses[7], [8] recommend not using loop diuretics to prevent AKI. To date, our knowledge on this issue is fairly well evidence-based. However, the role of osmotic diuretics in the prevention of AKI has not been well established. Mannitol, an osmotic diuretic, has been used in clinical practice for the prevention of AKI because of its potentially renal protective effects: removal of obstructing tubular casts, dilution of nephrotoxic substances in the tubular fluid, and reduction in the swelling of tubular elements via osmotic extraction of water.[9] Additionally, prophylactic mannitol is effective in animal models of AKI.[10], [11] On the other hand, potential nephrotoxicity of mannitol raised clinician's concerns. Mannitol may induce extensive isometric renal proximal tubular vacuolization, intense afferent arteriolar constriction (particularly when combined with cyclosporine A) and acute renal failure in higher doses.[12]–[14] The results of available clinical researches were also conflicting [15]–[17] and most of the studies are retrospective, underpowered and inconclusive. Due to the lack of strong evidence, the *KDIGO Clinical Practice Guideline for Acute Kidney Injury* published in 2012 did not propose completely evidence-based recommendations on this issue.[1] To answer the question whether mannitol use in high-risk patients can ameliorate renal outcomes and improve the prognosis, we carried out this systematic review and meta-analysis on the efficiency of using mannitol in patients with increased risk of AKI.

## Methods

The protocol of this research has been submitted.

### Search strategy and study selection

A search of the medical literature was conducted using PubMed (up to May 2013), EMBASE (1980 to May 2013), Cochrane Controlled Trials Register (issue 4, 2013) and the Clinical Trials Registry (http://clinicaltrials.gov/) (date of search: 2, May 2013). Studies on AKI were identified with the terms *acute kidney injury*; *renal failure, acute* and *mannitol* (either as medical subject heading (MeSH) and free text terms. These were combined using the set operator AND. We also searched the reference lists of the original reports, reviews, letters to the editor, case reports, guidelines and meta-analyses of studies involving mannitol and AKI (retrieved through the electronic searches) to identify studies, which had not yet been included in the computerized databases. All potentially relevant papers were obtained and evaluated in detail. There were no language restrictions. Articles were independently assessed by two review authors (BY and JX) using predesigned eligibility criteria: 1) Randomized Controlled Trials (RCTs); 2) Adult patients at risk for AKI, including contrast-induced AKI; 3) Comparing the effects of intravascular administration of mannitol plus expansion of intravascular volume to expansion of intravascular volume alone; 4) Providing data on renal outcomes. Trials using other pharmacotherapies, management of hemodynamic or oxygenation parameters were eligible, as long as these were administered to both the intervention and control groups. All doses of mannitol were considered. Where more than one publication of a trial existed, we used the most complete publication. We excluded trials with the following properties: 1) Enrolled patients undergoing any kinds of dialysis interventions; 2) Patients with volume overload who cannot tolerate expansion of intravascular volume; 3) Acute postrenal obstructive nephropathy; 4) Mannitol administrated via oral; 5) Any other interventions conducted only in the experimental group or in the control group; 6) No control group. We attempted to contact the original investigators in order to obtain further information if necessary. Any disagreement between review authors was resolved by consensus, and adjudicated with the support of a third review author (CY).

### Outcome assessment

The primary outcome assessed was change of serum creatinine concentration (SCr) (μmol/L). The secondary outcomes included incidence of renal failure or need of dialysis, and change of urine output (ml/24 h). Where more than one group of data were reported to monitor the progression of kidney injury, we selected the data collected 24 hours after the exposure of the external risk factors of AKI. For the recipients of kidney transplantation, we discarded the baseline SCr value, but extracted the change of SCr concentration between the moment after operation and the third day after operation.

### Data extraction

All data were extracted independently by two review authors (BY and JX.) to a predesigned form (Microsoft Office Excel 2007; Microsoft Corp, Redmond, Washington, USA). All data extraction was then checked by a third review author (CY). The following data were extracted for each trial: first author and publication year; number of centers; geographical location of the study; study population; sample size; proportion of female patients; risk factors of AKI (exposures and susceptibilities induced by comorbidities); interventions in the experimental and the control group; targeted dose; duration of study; concomitant medications; renal outcomes; outcome assessment during study; method used to generate the randomization schedule; allocation concealment and blinding. Data were extracted as intention-to-treat analyses, where all drop-outs were assumed to be treatment failures, wherever trial reporting allowed this. The exact mean and standard deviation (SD) may be difficult to decipher in some studies in which results are presented in figures (not tables). In this situation, two review authors independently estimated the exact values presented in the figures in each study using Engauge Digitizer 4.1 and achieve an agreement on the mean ± SD.

### Assessment of risk of bias

Assessment of risk of bias was performed independently by two review authors (BY and JX), with disagreements resolved by discussion. Risk of bias was assessed according to the quality domains of random sequence generation, allocation concealment, blinding of participants and personnel, blinding of outcome assessment, incomplete outcome data, selective reporting and any other potential threats to validity.[18]–[21] Risk of bias for each domain was rated as high (seriously weakens confidence in the results), low (unlikely to seriously alter the results), or unclear, as reported in the ‘Risk of bias’ table. A ‘Risk of bias summary’ figure which details all of the judgments made for all included studies in the review was generated.[18], [22]

### Data synthesis and statistical analysis

Heterogeneity among studies was assessed using the I^2^ statistic and χ^2^ test (assessing the P value). If the P value was less than 0.10 and I^2^ exceeded 50%, we considered heterogeneity to be substantial. Random effects model was used to combine the data if significant heterogeneity existed (P<0.1; I^2^>50%). Dichotomous data were summarized as risk ratio (RR); numbers needed to treat (NNT) and continuous ones as mean difference (MD), along with 95% confidence intervals (CIs), respectively. For continuous data, especially, when the mean values and SDs from the baseline to the point of data collecting were reported, they were retrieved directly. When standard errors (SEs) were reported instead of SDs, SDs were calculated using the formula: SD = SE*(n) ^0.5^.[18] If the mean values and SDs were not available, we computed them according to the Cochrane Handbook for Systematic Reviews of Interventions (version 5.1.0).[18]

We conducted prespecified subgroup analyses according to the various risk factors of AKI, including exposures (for example, cardiac surgery with cardiopulmonary bypass, major noncardiac surgery, radiocontrast agents and nephrotoxic drugs) and susceptibilities (such as chronic kidney disease and diabetes mellitus). Sensitivity analyses were planned to assess effects after removal of outlier RCTs identified in funnel plots. These were exploratory analyses only, and may explain some of the observed variability. The results, however, should be interpreted with caution.

Review Manager (RevMan) [Computer program]. Version 5.2. (Copenhagen: The Nordic Cochrane Centre, The Cochrane Collaboration, 2012) was used to generate forest plots for outcomes with 95% CIs, as well as funnel plots. The funnel plots were assessed for evidence of asymmetry, and possible publication bias or other small study effects.

## Results

**Figure 1:**
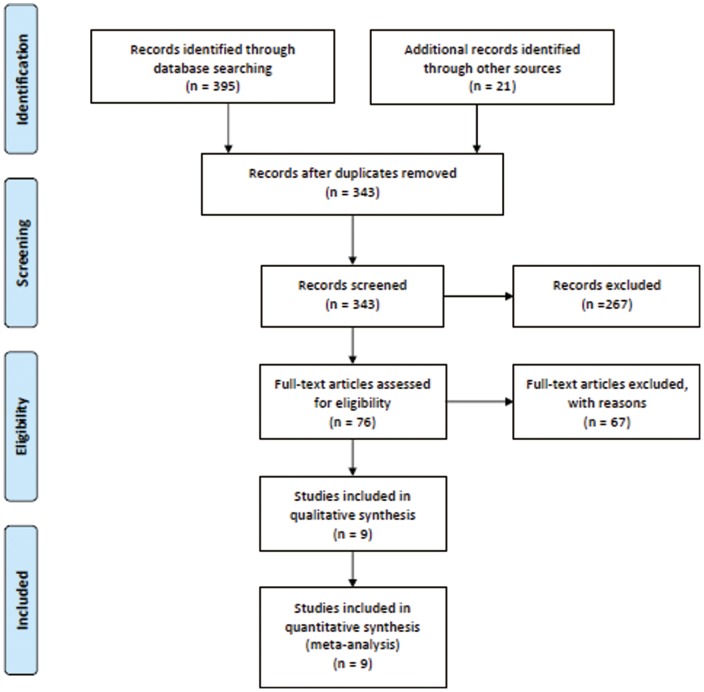
Flow diagram of literature search and study selection. RCT, randomized controlled trial

The search strategy initially yielded 416 citations, 76 of which appeared to be relevant to the systematic review and were retrieved for further assessment.(Figure 1) Of these, 67 were excluded for various reasons, leaving a total of nine eligible articles.[23]–[31] Among the RCTs included, two studies[26], [27] contain multiple but no shared intervention groups. We split these two trials into two pairs of eligible comparisons, respectively.

### Study characteristics

Table 1 summarizes the characteristics of the included studies. The nine RCTs enrolled 626 adult patients at increased risk of AKI. 262 patients in four trials[25], [27], [29], [30] underwent elective cardiac surgery with CPB[27], [29], [30] or major noncardiac surgery.[25] 128 participants in two RCTs[24], [28] received radiocontrast agents, which had pre-existing renal dysfunction (SCr>140 µmol/L). Gender of participants in three RCTs was unavailable.[24], [26], [31] One study[23] containing 55 female subjects focused on the patients prescribed nephrotoxic drug (cisplatin), while in the other five trials,[25], [27]–[30] study populations were male-dominated (73.0%). Two trials[26], [31] studied 181 recipients of a cadaveric renal allograft. Except for the 181 recipients of a renal allograft, another 178 patients in three RCTs[24], [28], [30] already have pre-existing renal dysfunction. The targeted dose of mannitol in experimental groups were fixed in five trials[23], [24], [26], [28], [31] (range 25 g to 50 g), while in the other four studies, mannitol was administrated according to body weight of the subjects (range 0.3 g/kg to 1 g/kg). The control management in each trial is expansion of intravascular volume using crystalloid fluid (normal saline, Hartmann's solution, etc.). Renal outcomes were reported in each trial, including SCr, creatinine clearance (CCr), plasma urea, urinary volume and need of dialysis or acute renal failure, but the definition of acute renal failure varied across studies, and it was not clear in one study.[26] No clear and definite adverse events can be identified in any of the trials.

**Table 1:** Table of characteristics of included studies.

Study	Study population	Mean age (years)	Sample size (male)	Interventions	Outcomes	Outcome assessment during study
**Carcoana 2003** [**27**]	SCr level of <1.5 mg/dL, undergoing elective, primary CABG surgery with CPB	63.3; 64.3; 63.8; 63.4	24(18); 26(19); 25(17); 25(18)	1) placebo, 2) mannitol 1 g/kg added to the CPB prime, 3) DA 2 µg/(kg*min) from the induction of anesthesia to 1 h post-CPB, 4) mannitol plus DA.	β_2_M excretion rate at 1 h post-CPB; β_2_M excretion rates at 6 and 24 h post-CPB; creatinine clearance; postoperative serum creatinine levels; Urinary output; Length of ICU stay; length of hospitalization; significant clinical events	Urinary output was noted hourly before and after CPB; β_2_M excretion rate at 1, 6, and 24 h after CPB; highest postoperative serum creatinine was recorded
**Nicholson 1996** [**25**]	Patients undergoing elective aortic aneurysm repair surgery	68; 71	15(13); 13(11)	receive either 1) mannitol 0.3g/kg or 2)an equivalent volume of normal saline given as a rapid intravenous infusion before cross-clamping the aorta.	urine output; creatinine clearance; blood urea; acute renal failure; serum creatinine; urinary albumin; urinary N-acetylglucosaminidase; urinary creatinine	6 h; 24 h; 3d and 7d after surgery
**Santoso 2003** [**23**]	be to receive 75 mg/m^2^ of cisplatin alone or in combination with paclitaxel or 5-fluorouracil to treat gynecologic cancers.	49.0; 48.0; 43.7	17(0); 19(0); 19(0)	1)500 mL NS in 2 h and mix cisplatin in 1 L NS; 2)500 mL NS in 2 h and mix cisplatin in 1 L NS with 50 g mannitol; 3) 500 mL NS in 2 h, 40 mg furosemide 30 min before cisplatin and mix cisplatin in 1 L NS	24 h creatinine clearance; SCr; metabolic panel; CA-125 for patients with ovarian cancer	Each clinic visit after cisplatin
**Smith 2008** [**30**]	Adult patients having elective cardiac surgery with CPB, and pre-operative renal dysfunction: 130 µmol/L< SCr < 250 µmol/L	74.7; 74.7	23(16); 24(18)	1) 0.5 g/kg of mannitol as a 20% solution in the prime. 2) equivalent volume of Hartmann's solution	Daily urine output and plasma creatinine and urea	1, 2, 3d after surgery
**Solomon 1994** [**28**]	Patients scheduled for cardiac angiography who had SCr >140 µmol/L or CCr<60 ml/min	67; 60; 63	28(23); 25(19); 25(12)	1) 0.45% NS 1 ml/(kg*h) 2) 0.45% NS 1 ml/(kg*h)+ 25 g mannitol, infused intravenously during the 60 minutes immediately before angiography. 3) 0.45% NS 1 ml/(kg*h)+80 mg furosemide, infused intravenously during the 30 minutes immediately before angiography.	SCr; BUN; urinary sodium; urinary potassium; urinary creatinine	At the time of angiography, 1d,2d
**van Valenberg 1987** [**26**]	Recipient of a cadaveric renal allograft	NA	33; 34; 32; 32. Gender: NA	Azathioprine: 1) 20% mannitol 250 ml 2) 5% glucose 250 ml during the last 10 min before the opening of the vascular anastomoses. Cyclosporine: 1) 20% mannitol 250 ml 2) 5% glucose 250 ml during the last 10 min before the opening of the vascular anastomoses.	ARF; SCr postoperatively; graft survival; patient survival	SCr: 1, 2, 3d; graft/patient survival: 3m, 1y
**Weimar 1983** [**31**]	Recipient of a cadaveric renal allograft	Donor:21.5; 20.5. Recipient: 34; 38	22; 22. Gender: NA	1) 20% mannitol 250 ml i.v. before revascularization 2) NS	Patients with immediate renal function; CCr; patients with ATN leading to dialysis	NA
**Weisberg 1984** [**24**]	SCr> 1.8 mg/dl, undergoing elective cardiac catheterization	NA	15; 15; 10; 10. Gender: NA	1) saline 100 ml/h; 2) dopamine 2 µg/(kg*min) in NS, 100 ml/h; 3) ANP 50 µg bolus, followed by an infusion of 1 µg/min in NS,100 ml/h; 4) mannitol 15 g/dl in NS 100 ml/h. the infusions began immediately after full instrumentation for the cardiac catheterization procedure and continued for a total of two hours	RBF; SCr	SCr: 1d, 2d. RBF: baseline, after the drug infusion was begun but before the ventriculogram, immediately after the ventriculogram, after the coronary angiogram
**Yallop 2008** [**29**]	Patients scheduled for elective cardiac surgery with CPB	64.1; 62.2	20(14); 20(16)	1) 5 ml/kg of 10% mannitol in the pump prime. 2) Hartmann's solution in the pump prime	Urinary creatinine; microalbumin; SCr; plasma urea; urine output	1, 2, 3, 4, 5d

Abbreviations: NA, not applicable; SCr, serum creatinine; CABG, coronary artery bypass surgery; CPB, cardiopulmonary bypass; DA, dopamine; ICU, intensive care unit; NS, normal saline solution; RBF, renal blood flow; BUN, Blood urea nitrogen; CCr, creatinine clearance; ATN, acute tubular necrosis.

### Risk of bias

Risk of bias ratings for each trial (Table 2, Figure S1) were assessed with the Cochrane risk of bias tool.[22] In the domain of random sequence generation, six RCTs were at low risk of bias,[23], [25], [27]–[30] while three trials declared as “randomized” but did not report the method of randomization (unclear risk of bias).[24], [26], [31] No RCTs were at high risk of bias for allocation concealment, however, the method of concealment was unclear (not reported) in five trials.[23], [24], [26], [28], [31] None of the studies reported the blinding of outcome assessment, which result in an unclear detection risk of bias in each of the included trials. Additionally, only three RCTs provided the information of blinding of participants and personnel.[24], [27], [29] In one trial,[26] a high risk of bias was identified in domain of incomplete outcome data. The investigators of this RCT provided SCr levels in cyclosporine-treated patients without acute renal failure who received either mannitol or 5% glucose, but the patients with acute renal failure were excluded from the analysis. Numbers excluded are not balanced across groups, which may introduce attrition bias. According to the data provided in this original study, we can also recognize the direction of bias, which favors the group prescribed 5% glucose. In the trial conducted by Nicholson, we are not clear whether the patients developing postoperative complications were excluded from the analysis,[25] so we graded the study as unclear risk of bias in this domain. We found no suspect selective reporting in all of the included RCTs.

**Table 2:** Risk of bias.

Study	Random sequence generation	Allocation concealment	Blinding of participants and personnel	Blinding of outcome assessment	Incomplete outcome data	Selective reporting
**Carcoana 2003** [**27**]	Computer-generated random-number tables	Patients were randomly allocated by the Department of Investigational Pharmacy	double-blinded	NA	No drop-outs after randomization	The results of all outcomes described in methods were reported
**Nicholson 1996** [**25**]	Table of random numbers	Sealed envelope	NA	NA	Unclear bias	The results of all outcomes described in methods were reported
**Santoso 2003** [**23**]	Randomized allocation table	NA	NA	NA	Non-intention-to-treat analysis	The results of all outcomes described in methods were reported
**Smith 2008** [**30**]	computer-generated random number tables	the anesthetic, theatre and ICU staff were blind to the randomization	NA	NA	No drop-outs after randomization	The results of all outcomes described in methods were reported
**Solomon 1994** [**28**]	Random-allocation table	NA	NA	NA	No drop-outs after randomization	The results of all outcomes described in methods were reported
**van Valenberg 1987** [**26**]	Randomized controlled trial	NA	NA	NA	Patients with ARF were not analyzed	The results of all outcomes described in methods were reported
**Weimar 1983** [**31**]	Random allocated	NA	NA	NA	Intention-to-treat analysis	The results of all outcomes described in methods were reported
**Weisberg 1984** [**24**]	Randomized controlled trial	NA	Double-blind	NA	No drop-outs after randomization	The results of all outcomes described in methods were reported
**Yallop 2008** [**29**]	computer-generated random number chart	All personnel other than the perfusionist were blinded to the randomization	double-blind	NA	No drop-outs after randomization	The results of all outcomes described in methods were reported

Abbreviations: NA, not applicable; ICU, intensive care unit.

### Effects of interventions

#### Serum creatinine

**Figure 2:**
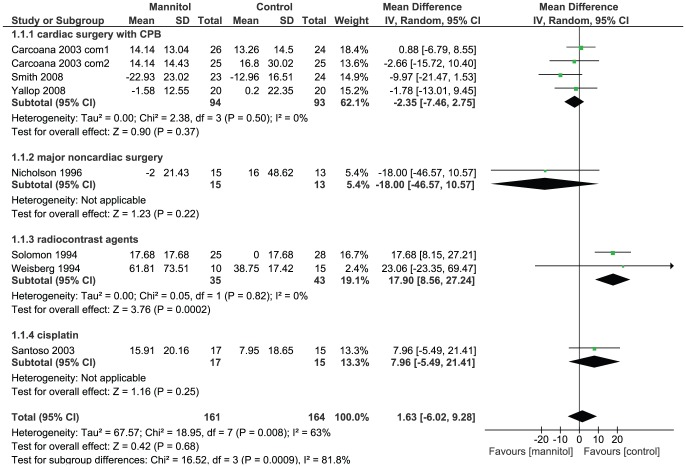
Change of serum creatinine level among participants given mannitol versus control. Note that Carcoana 2003 contain multiple but no shared intervention groups. We split it into two pairs of eligible comparisons (Carcoana 2003 com1 and Carcoana 2003 com2)

Eight studies reported the outcome of SCr change. Overall, there was no significant difference between the experimental group and control group (MD 1.63, 95% CI −6.02 to 9.28; I^2^ = 63%, P = 0.008). Statistically non-significant results were identified in three of the subgroup analyses according to the potential etiologies of AKI: 1) cardiac surgery with CPB (MD −2.35, 95% CI −7.46 to 2.75; I^2^ = 0%, P = 0.50); 2) major noncardiac surgery (MD −18.00, 95% CI −46.57 to 10.57) and 3) nephrotoxic drugs (MD 7.96, 95% CI −5.49 to 21.41). In the subgroup of radiocontrast agents, a greater increase of SCr in mannitol groups was found (MD 17.90, 95% CI 8.56 to 27.24; I^2^ = 0%, P = 0.82), which means the administration of mannitol may exacerbate AKI in patients undergoing radiocontrast agents injection.(Figure 2) Patients in three trials[24], [28], [30] already had pre-existing renal dysfunction, when data of these three RCTs were combined exclusively, no significant difference between mannitol groups and control groups was found (MD 7.18, 95% CI −16.29 to 30.66; I^2^ = 85%, P = 0.001). There is also a study focusing on recipients of a cadaveric renal allograft provided the level of SCr, but the result of this trial was not combined with other RCTs', due to the obvious clinical heterogeneity. Analysis of this orphan study shows that compared with the control group, SCr level did not decrease significantly in the mannitol group (MD −141.46, 95% CI −284.93 to 2.01). However, the upper bound of 95% CI is close to “0”, which means the results should be interpreted with caution. (Figure S2)

#### Acute renal failure or need of dialysis

**Figure 3:**
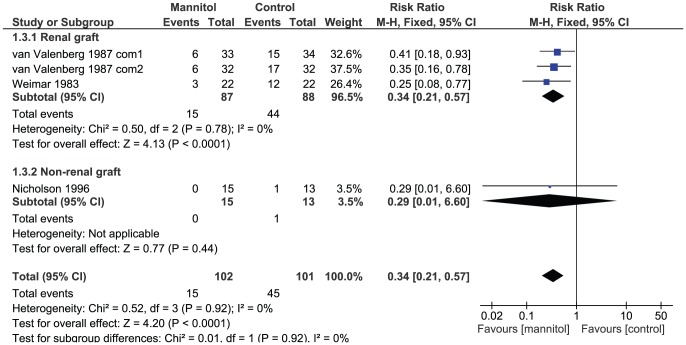
Risk of acute renal failure or need of dialysis intervention among participants given mannitol versus control. Note that van Valenberg 1987 contains multiple but no shared intervention groups. We split it into two pairs of eligible comparisons (van Valenberg 1987 com1 and van Valenberg 1987 com2)

Five comparisons in four studies reported the acute renal failure or need of dialysis. (Figure 3) The overall result indicates that mannitol administration may reduce the incidence of acute renal failure or reduce the need of dialysis (RR 0.34, 95% CI 0.21 to 0.57, NNT 3.45, 95% CI 2.44 to 5.56; I^2^ = 0%, P = 0.92), but the statistically significant result is stable only in the subgroup of renal graft (RR 0.34, 95% CI 0.21 to 0.57, NNT 3.03, 95% CI 2.17 to 5.00; I^2^ = 0%, P = 0.78). In the subgroup of non-renal graft, we identified no difference between interventions and controls (RR 0.29, 95% CI 0.01 to 6.60).

#### Urine output

**Figure 4,:**
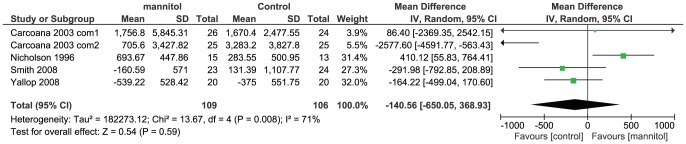
Change of urine output among participants given mannitol versus control. Note that Carcoana 2003 contains multiple but no shared intervention groups. We split it into two pairs of eligible comparisons (Carcoana 2003 com1 and Carcoana 2003 com2)

Change of urinary volume can be extracted from five comparisons in four RCTs. (Figure 4) There was no difference in change of urine output (MD −140.56, 95% CI −650.05 to 368.93), but the heterogeneity was significant across studies (I^2^ = 71%, P = 0.008). The funnel plot was generated (not shown), and data in two comparisons from one study[27] were identified as outliers. Then a planned sensitivity analysis was carried out by excluding these two comparisons and the result was stable in sensitivity analysis (MD 2.07, 95% CI −428.54 to 432.67; I^2^ = 73%, P = 0.03). (Figure S3)

#### Publication bias

No evidence of publication bias for the primary outcome was indicated by visual inspection of the funnel plots (not shown). The effect of an outlying study[27] on the outcome of urine output was assessed with a sensitivity analysis. As the results mentioned above, removal of this study did not change the primary result.

## Discussion

### Summary of results and possible explanations

This systematic review and meta-analysis included nine trials with 626 participants at increased risk of AKI involved. All RCTs were carried out in hospital setting, where the patient's risk factors can be assessed before certain exposures. The patients enrolled were exposed to several common risks of AKI, and this heterogeneity provided a good representativeness of clinical practice. Since the methodological qualities of these studies were relatively high, and no publication bias was identified, we considered the quality of evidence is good and the true effect lies close to that of the estimate of the effect. Our results demonstrated that intravascular administration of mannitol for AKI prevention in high-risk patients can not ameliorate the deterioration of renal function. Moreover, SCr level is negatively affected by the use of mannitol in patients undergoing radiocontrast agents injection. In other words, prophylactic mannitol in this kind of patients may be associated with significant toxicity. Although in some animal researches, mannitol provides beneficial effects against contrast-induced nephropathy,[32] the present meta-analysis and former studies based on human concluded the opposite.[33], [34] On the other hand, recipients of a cadaveric renal allograft may benefit from the use of mannitol before vessel clamp removal. Recipients prescribed mannitol may experience a greater SCr decrease, smaller chance of acute renal failure and fewer dialysis interventions after transplantation. However, the two included studies[26], [31] focusing on the renal transplantation were both conducted in 1980s, and surgical technic, immunosuppressive therapies and other risk factors of AKI have evolved significantly since then.[35], [36] Pooling data in the early days alone will damage the completeness and applicability of evidence, and the above results should be interpreted with caution.

The lack of a significant diuretic effect when receiving mannitol is interesting, as showed in this analysis, which seems to be inconsistent with well established knowledge.[37] The first issue to be addressed is clinical heterogeneity. In the trial conducted by Carcoana,[27] urinary output was noted hourly, while in other three RCTs, it was recorded daily. This makes the unit of measurement across studies different (ml/min *vs.* ml/24 h). After the conversion of the ml/min into ml/24 h,[18] heterogeneity (especially in SD value) showed up. The clinical heterogeneity may comes from this kind of nondifferential measurement bias. After excluding this study, we found the result was stable.

Clinically, mannitol has been used to treat fluid overload and cerebral edema. The diuretic effect of mannitol in these non-AKI risk patients was affirmed (although the benefit of its clinical use in decreasing the intracranial pressure is under estimation).[38], [39] But according to the results of the present study, the diuretic effect of mannitol was significantly weakened in the patients with increased risk of AKI. It is reasonable to regard the diuretic response as a predictor of renal outcome rather than the therapeutic effect for patients with AKI risk.[40] As four out of five pairs of comparisons in this outcome studied patients undergoing cardiac surgery with CPB, another possible explanation can be reasonable that renal dysfunction after CPB may be induced by micro emboli, in which case mannitol is unlikely to be of diuretic benefit.[41]

### Results in relation to other studies

So far, no published systematic review or meta-analyses have assessed the efficiency of mannitol for AKI prevention. A conventional review on this clinical issue published in 2004 summarized the retrospective studies and clinical trials before the year of 2000.[42] In this review, the authors concluded that mannitol has not been proven to be of value for renal protection in humans. This conclusion is consistent with ours, but available evidence at that time was largely underpowered. We included the recently completed well-designed RCTs (Figure S1) and enhanced the strength of the evidence. In accordance with our results, another narrative review of the literature studying the perioperative fluid management in renal transplantation found salutary effects of mannitol infusions in kidney transplantation immediately before opening the vascular anastomoses.[43] The reviewers summarized the results of animal researches, retrospective studies, as well as clinical trials. Due to the nature of narrative review, no clear inclusion criteria and no risk of bias assessment was applied in this study, we considered the strength of the conclusion in that review very low.

### Strengths and limitations

Our systematic review and meta-analysis has several strengths. Firstly, most of the trials included in the meta-analysis were of good methodological quality, (Figure S1) which makes the results of meta-analysis less likely to be affected by the biases of the original studies. Secondly, the populations studied varied widely and covered several major risk factors of AKI. Including such a heterogeneous group may increase the generalizability of our review. In addition, we performed appropriate subgroup analyses which fit for investigating heterogeneous results. Finally, since the definition of acute renal failure varied across studies, we chose SCr rather than acute renal failure or need of dialysis as our primary outcome. This design may estimate the efficiency of interventions more exactly.

Our research also has several limitations. First, few RCTs set mortality as their endpoint, which may not be useful when we assess the association between interventions and prognosis. Surrogate endpoints do not always translate into prognosis. Besides, since AKI is a risk factor for chronic kidney disease, it is important to evaluate the long-term renal function of enrolled patients. But the durations of follow-up in these included studies were relatively short. Only an individual study we included reported the renal function after three months and one year of exposure of AKI risk factor (renal transplantation).[26] The result of this original trial was that the mannitol-induced reduction in the incidence of acute renal failure had no impact on patient or graft survival. In addition, no clear and definite adverse events were reported in any of the RCTs, it is understandable because in this situation, many signs and symptoms can be attributed to either drug side effects or the impaired renal function. Distinguishing these clinical manifestations clearly was impractical.

### Conclusions and implications for future research

Intravascular administration of mannitol does not convey additional beneficial effects beyond adequate hydration in the patients at increased risk of AKI. Its use for AKI prevention is not scientifically justified, and for contrast-induced nephropathy prevention is even detrimental. The findings of this review suggest that further research evaluating efficiency of mannitol infusions in the recipients of renal allograft should be undertaken. Besides, the endpoints in studies involved were set as immediate renal function; SCr; and acute renal failure, long term graft and patient survival, and function index should be investigated.

## Supporting Information

Figure S1 **Risk of bias**
(EPS)

Figure S2 **Change of serum creatinine level among renal graft recipients given mannitol versus control.** Note that van Valenberg 1987 contains multiple but no shared intervention groups. We split it into two pairs of eligible comparisons (van Valenberg 1987 com1 and van Valenberg 1987 com2)
(EPS)

Figure S3 **Sensitivity analysis of change of urine output among participants given mannitol versus control.** Note that Carcoana 2003 contains multiple but no shared intervention groups. We split it into two pairs of eligible comparisons (Carcoana 2003 com1 and Carcoana 2003 com2)
(EPS)

Checklist S1 **PRISMA checklist.**
(DOC)

Protocol S1 **Study protocol.**
(DOC)
