# Supplementary material for: Intravascular Administration of Mannitol for Acute Kidney Injury Prevention: A Systematic Review and Meta-Analysis
Source: PLoS One. 2014 Jan 14;9(1):e85029. doi: 10.1371/journal.pone.0085029 (PMC3891750; doi:10.1371/journal.pone.0085029)
Supplement: Protocol S1 — Study protocol. (DOC) [file pone.0085029.s005.doc]

**Intravascular administration of mannitol for acute kidney injury prevention: systematic review and meta-analysis of randomised controlled trials (protocol)**

**Type of article:** Original Article

**Authors:** Bo Yang, Jing Xu, Chaoyang Ye , Changlin Mei, Zhiguo Mao

The first 2 authors contributed equally to this work.

**Institution and Affiliations:**

Bo Yang, Graduate Student, Kidney institute of CPLA, Division of Nephrology, Changzheng hospital, Second Military Medical University, Shanghai, China

Jing Xu, Attending Doctor, Kidney institute of CPLA, Division of Nephrology, Changzheng hospital, Second Military Medical University, Shanghai, China

Chaoyang Ye, Professor, Kidney institute of CPLA, Division of Nephrology, Changzheng hospital, Second Military Medical University, Shanghai, China

Changlin Mei, Professor, Kidney institute of CPLA, Division of Nephrology, Changzheng hospital, Second Military Medical University, Shanghai, China

Zhiguo Mao, Associate Doctor, Kidney institute of CPLA, Division of Nephrology, Changzheng hospital, Second Military Medical University, Shanghai, China

**Correspondence to:**

* Zhiguo Mao, Kidney institute of CPLA, Division of Nephrology, Changzheng hospital, Second Military Medical University, 415 Fengyang Road, Shanghai 200003, People’s Republic of China. Phone: +86-21-81885394; Fax: +86-21-81885411; E-mail address: maozhiguo93@gmail.com

* Changlin Mei, Kidney institute of CPLA, Division of Nephrology, Changzheng hospital, Second Military Medical University, 415 Fengyang Road, Shanghai 200003, People’s Republic of China. Phone: +86-21-81885394; Fax: +86-21-81885411; E-mail address: chlmei1954@126.com

**ABSTRACT**

This is the protocol for a review.

The objective of this review is to determine whether intravascular administration of mannitol for patients at high risk of acute kidney injury (AKI) can ameliorate renal outcomes.

**BACKGROUND**

**Description of the condition**

Acute kidney injury (AKI) is defined as an abrupt decrease in renal function. It is a broad clinical syndrome encompassing various aetiologies, including sepsis, dehydration, cardiac surgery (especially with cardiopulmonary bypass(CPB)), radiocontrast agents and so on. Many analytical studies suggest that AKI is associated with prolonged length of stay, increased mortality, and increased in-hospital and posthospitalisation resource utilisation. Based on the relatively adverse clinical consequences and high health-care costs of AKI, early recognition and management of the patients at high risk of AKI is paramount.

**Description of the intervention**

Intravascular mannitol has been frequently used for prevention of AKI. The targeted dose of mannitol in experimental groups can be fixed, or can be adjusted according to the body weight of the patients. In the situation of renal transplantation, mannitol is usually infused before vessel clamp removal. In the patients undergoing cardiac surgery with CPB, mannitol can be added to the CPB prime.

**How the intervention might work**

Mannitol is freely filtered at the glomerulus, undergo limited reabsorption by the renal tubule, and is relatively inert pharmacologically. It acts primarily at the loop of Henle and also on the proximal tubule by extracting water from intracellular compartments. Mannitol expands the extracellular fluid volume, decreases blood viscosity, and inhibits renin release. These effects generally result in an increase in renal blood flow and a reduction in medullary tonicity. The renal protection afforded by mannitol may be due to the removal of obstructing tubular casts, dilution of nephrotoxic substances in the tubular fluid, and/or reduction in the swelling of tubular elements via osmotic extraction of water. In addition, mannitol has also been shown to increase RBF and to act as a free-radical scavenger during reperfusion of the kidney.

**Why it is important to do this review**

The conclusions of many clinical researches were conflicting, and most of the studies are retrospective, underpowered and inconclusive. Due to the lack of strong evidence, the *KDIGO Clinical Practice Guideline for Acute Kidney Injury* published in 2012 did not propose any evidence-based recommendations on this issue as well.

**OBJECTIVES**

The objectives of this review are to determine whether intravascular administration of mannitol for patients at high risk of acute kidney injury (AKI) can ameliorate renal outcomes.

**METHODS**

**Criteria for considering studies for this review**

***Types of studies***

Randomised controlled trials.

***Types of participants***

We will include:

Adult patients at risk for AKI, including contrast-induced AKI.

We will exclude:

1. Enrolled patients undergoing any kinds of dialysis interventions;
2. Patients with volume overload who can not tolerate expansion of intravascular volume;
3. Acute postrenal obstructive nephropathy.

***Types of interventions***

We will include:

Comparing the effects of intravascular administration of mannitol plus expansion of intravascular volume to expansion of intravascular volume alone.

We will exclude:

1. Mannitol administrated via oral;
2. Any other interventions conducted only in experimental group or in control group;
3. No control group.

***Types of outcome measures***

We will include the following outcomes:

1. Renal outcomes (serum creatinine; creatinine clearance; urinary output; need of dialysis; acute renal failure; proteinuria value; etc.);
2. Long-term renal function;
3. Adverse effects;
4. Renal dysfunction related mortality.

**Search methods for identification of studies**

***Electronic searches***

We will search PubMed (up to May 2013), EMBASE (1980 to May 2013), Cochrane Controlled Trials Register (issue 4, 2013) and the Clinical Trials Registry (http://clinicaltrials.gov/). Studies on AKI will be identiﬁed with the terms *acute kidney injury*; *renal failure, acute* and *mannitol* (either as medical subject heading (MeSH) and free text terms. These will be combined using the set operator AND. Similar search terms will be used for EMBASE.

***Searching other resources***

We will also search the reference lists of the original reports, reviews, letters to the editor, case reports, guidelines and meta-analyses of studies involving mannitol and AKI (retrieved through the electronic searches) to identify studies which have not yet been included in the computerised databases.

*Unpublished studies*

We will not consider unpublished trial data.

*Language*

We will apply no restrictions with respect to language.

**Data collection and analysis**

***Selection of studies***

Two review authors will independently assess the eligibility of a study to be included in the review, and this will be checked by another review author. If a study is reported several times and some data are repeated, we will only include the most complete version to avoid duplication. We will resolve any disagreement by discussion.

***Data extraction and management***

Two review authors will independently extract data from each identified study and record data on a standardised data extraction form (appendix 1). We will resolve any disagreement by discussion.

***Assessment of risk of bias in included studies***

Two review authors will assess the included studies independently, based on the quality domains of random sequence generation, allocation concealment, blinding of participants and personnel, blinding of outcome assessment, incomplete outcome data, selective reporting and any other potential threats to validity. Risk of bias for each domain will be rated as high (seriously weakens confidence in the results), low (unlikely to seriously alter the results), or unclear. We will report the 'Risk of bias' table as part of the 'Table of characteristics of included studies'. A 'Risk of bias summary' figure which details all of the judgements made for all included studies in the review will be generated.

To avoid selection bias, we will include all studies, regardless of high or low risk of bias.

***Dealing with missing data***

We will contact the corresponding author of included trials to obtain missing data necessary for meta-analysis. If we fail to get reply of the author, the corresponding trial will be excluded. When standard errors (SEs) are reported instead of SDs, SDs will be calculated using the formula: SD = SE*(n)^0. 5^. If the mean values and SDs are not available, we computed them according to the Cochrane Handbook for Systematic Reviews of Interventions (version 5.1.0). The exact mean and standard deviation (SD) may be difficult to decipher in some studies in which results are presented in figures (not tables). In this situation, two review authors will independently estimate the exact values presented in the figures in each study using Engauge Digitizer 4.1 and achieve an agreement on the mean ± SD.

***Assessment of heterogeneity***

Heterogeneity among studies will be assessed using the I^2^ statistic and χ^2^ test (assessing the P value). If the P value is less than 0.10 and I^2^ exceed 50%, we will consider heterogeneity to be substantial. Random effects model will be used to combine the data if significant heterogeneity existed (P < 0.1; I^2^ > 50%).

***Data synthesis***

*Quantitative analysis*

We plan a statistical analysis of outcomes. We will merge both dichotomous and continuous data quantitatively in meta-analysis if the data are sufficient. Review Manager (RevMan) [Computer program]. Version 5.2. (Copenhagen: The Nordic Cochrane Centre, The Cochrane Collaboration, 2012) was used to generate forest plots for outcomes with 95% confidence intervals (CIs).

*Dichotomous data*

Dichotomous data were summarised as risk ratio (RR); numbers needed to treat (NNT), along with 95% CIs.

*Continuous data*

We will undertake a statistical analysis to deal with continuous data, and continuous ones as mean difference (MD), along with 95% CIs.

***Assessment of risk of publication bias***

The funnel plots will be assessed for evidence of asymmetry, and possible publication bias or other small study effects.

**Subgroup analysis and investigation of heterogeneity**

We will conduct a prespecified subgroup analyses according to the various risk factors of AKI, including exposures (for example, cardiac surgery with cardiopulmonary bypass, major noncardiac surgery, radiocontrast agents and nephrotoxic drugs) and susceptibilities (such as chronic kidney disease and diabetes mellitus).

**Sensitivity analysis**

Sensitivity analyses were planned to assess effects after removal of outlier RCTs iderntified in funnel plots. These are exploratory analyses only, and may explain some of the observed variability. The results, however, should be interpreted with caution.

**APPENDICES**

**Appendix 1: standardised data extraction form**

| Study |  |
| --- | --- |
| Study population |  |
| Mean age (years) |  |
| Sample size (male) |  |
| Interventions |  |
| Outcomes |  |
| Outcome assessment during study |  |
| Random sequence generation |  |
| Allocation concealment |  |
| Blinding of participants and personnel |  |
| Blinding of outcome assessment |  |
| Incomplete outcome data |  |
| Selective reporting |  |

Deviations from the protocol

1. Results of renal dysfunction related mortality, adverse events and long-term renal function in individual RCTs were not possible to be combined because total adverse events data were sparse.
2. We rearrange the subgroup analyses and sensitivity analyses to make them more clear to the editors, reviewers and readers.
3. New authors joined our team.

**REFERENCES**

1. Kidney Disease: Improving Global Outcomes (KDIGO) Acute Kidney Injury Work Group. KDIGO Clinical Practice Guideline for Acute Kidney Injury. *Kidney Int* 2012; Suppl. 2:1-138.

2. Harel Z, Chan CT. Predicting and preventing acute kidney injury after cardiac surgery. *Curr Opin Nephrol Hypertens* 2008; 17:624-628.

3. Venkataraman R. Can we prevent acute kidney injury? *Crit Care Med* 2008; 36:S166-171.

4. Coca SG, Yusuf B, Shlipak MG *et al*. Long-term risk of mortality and other adverse outcomes after acute kidney injury: a systematic review and meta-analysis. *Am J Kidney Dis* 2009; 53:961-973.

5. Xue JL, Daniels F, Star RA *et al*. Incidence and mortality of acute renal failure in Medicare beneficiaries, 1992 to 2001. *J Am Soc Nephrol* 2006; 17:1135-1142.

6. Uchino S, Kellum JA, Bellomo R *et al*. Acute renal failure in critically ill patients: a multinational, multicenter study. *JAMA* 2005; 294:813-818.

7. Karajala V, Mansour W, Kellum JA. Diuretics in acute kidney injury. *Minerva Anestesiol* 2009; 75:251-257.

8. Higgins J, Green S: **Cochrane Handbook for Systematic Reviews of Interventions Version 5.1.0 [updated March 2011]**. In*.* The Cochrane Collaboration; 2011.

9. Kjaergard LL, Villumsen J, Gluud C. Reported methodologic quality and discrepancies between large and small randomized trials in meta-analyses. *Ann Intern Med* 2001; 135:982-989.

10. Schulz KF, Chalmers I, Hayes RJ *et al*. Empirical evidence of bias. Dimensions of methodological quality associated with estimates of treatment effects in controlled trials. *JAMA* 1995; 273:408-412.

11. Moher D, Pham B, Jones A *et al*. Does quality of reports of randomised trials affect estimates of intervention efficacy reported in meta-analyses? *Lancet* 1998; 352:609-613.

12. Higgins JP, Altman DG, Gotzsche PC *et al*. The Cochrane Collaboration's tool for assessing risk of bias in randomised trials. *BMJ* 2011; 343:d5928.
